# The draft genome assembly of the critically endangered *Nyssa yunnanensis*, a plant species with extremely small populations endemic to Yunnan Province, China

**DOI:** 10.46471/gigabyte.4

**Published:** 2020-08-01

**Authors:** Weixue Mu, Jinpu Wei, Ting Yang, Yannan Fan, Le Cheng, Jinlong Yang, Ranchang Mu, Jie Liu, Jianming Zhao, Weibang Sun, Xun Xu, Xin Liu, Radoje Drmanac, Huan Liu

**Affiliations:** ^1^ State Key Laboratory of Agricultural Genomics, BGI-Shenzhen, Shenzhen 518083, China; ^2^ BGI-Yunnan, BGI-Shenzhen, Kunming, 650106, China; ^3^ Forestry Bureau of Ruili, Yunnan Dehong, Ruili 678600, China; ^4^ Yunnan Key Laboratory for Integrative Conservation of Plant Species with Extremely Small Populations, Kunming Institute of Botany, Chinese Academy of Sciences, Kunming 650204, Yunnan, China; ^5^ Department of Biology, University of Copenhagen, DK-2100 Copenhagen, Denmark; ^6^ Complete Genomics Inc., 2904 Orchard Pkwy, San Jose, CA 95134, USA; ^7^ Guangdong Provincial Key Laboratory of Genome Read and Write, Shenzhen 518083, China

## Abstract

*Nyssa yunnanensis* is a deciduous tree species in the family Nyssaceae within the order Cornales. As only eight individual trees and two populations have been recorded in China’s Yunnan province, this species has been listed among China’s national Class I protection species since 1999 and also among 120 PSESP (Plant Species with Extremely Small Populations) in the *Implementation Plan of Rescuing and Conserving China’s Plant Species with Extremely Small Populations (PSESP)* (2011-2-15). Here, we present the draft genome assembly of *N. yunnanensis*. Using 10X Genomics linked-reads sequencing data, we carried out the *de novo* assembly and annotation analysis. The *N. yunnanensis* genome assembly is 1475 Mb in length, containing 288,519 scaffolds with a scaffold N50 length of 985.59 kb. Within the assembled genome, 799.51 Mb was identified as repetitive elements, accounting for 54.24% of the sequenced genome, and a total of 39,803 protein-coding genes were predicted.

With the genomic characteristics of *N. yunnanensis* available, our study might facilitate future conservation biology studies to help protect this extremely threatened tree species.

## Data Description

**Figure 1.**
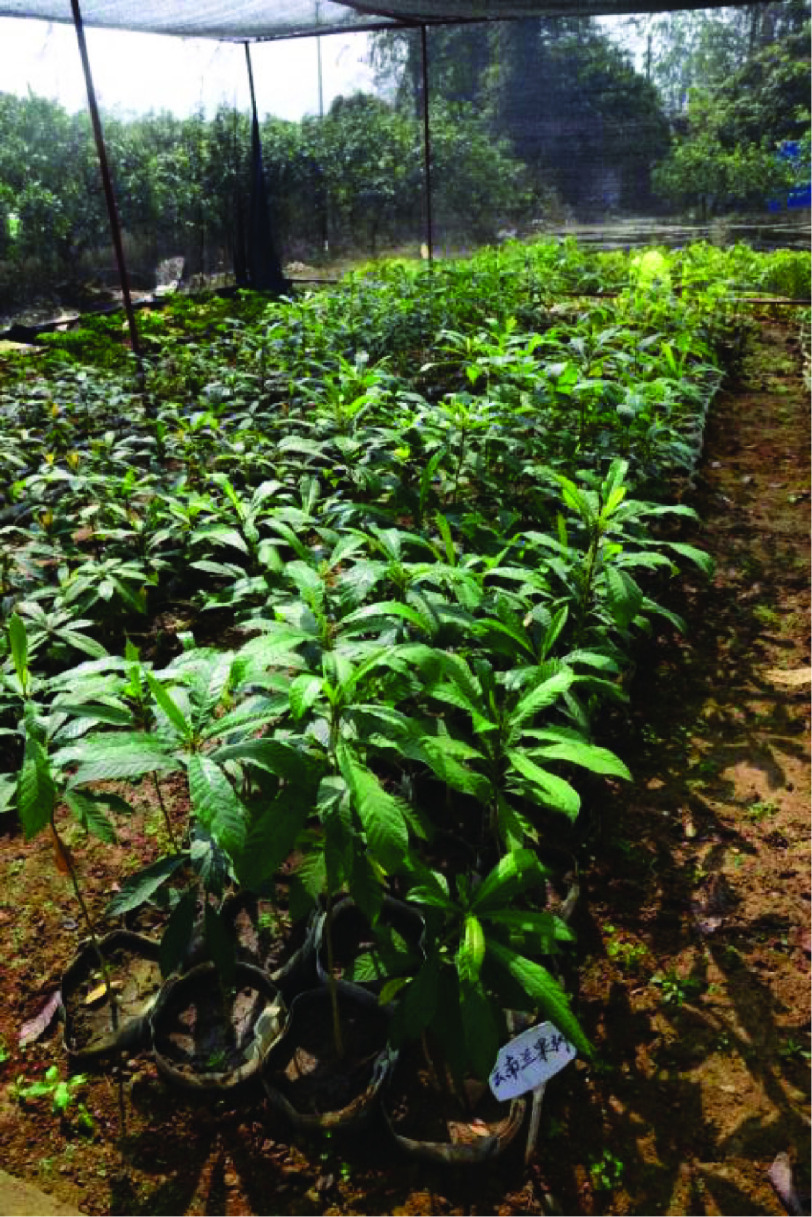
Photograph of *Nyssa yunnanensis* from Ruili, Yunnan Province, China.

*Nyssa yunnanensis* (NCBI: txid161873), which belongs to the family Nyssaceae, is an extremely threatened range-restricted tree species among the Critically Endangered (CR) in the IUCN Red List of Threatened Species [1], as well as a national key protected species under Class I protection in China [2]. *Nyssa yunnanensis* is also listed as one of 120 PSESP (Plant Species with Extremely Small Populations) in the *Implementation Plan of Rescuing and Conserving China’s Plant Species with extremely Small Populations (PSESP)* (2011-2-15) and as critically endangered in the Threatened Species List of China’s Higher Plants [3, 4] (Figure 1).

It is a canopy tree species able to reach 30 meters in height and it is functionally dioecious, consisting of two individual species. One species bears staminate flowers while the other type bears flowers that are morphologically normal but produce inaperturate and inviable pollen grains. *Nyssa yunnanensis* does not appear to exhibit parthenogenesis [5]. A survey of the population and ecological characteristics of *N. yunnanensis* suggested that this species is at high risk of extinction, as only two natural populations and eight individual trees have been recorded in Yunnan, China. This scarcity might be caused by both ecological and human factors [6]. In 2009, an integrated PSESP conservation strategy was initiated for *N. yunnanensis*. After more than seven years of implementation, the natural populations are now securely protected. Along with the development of propagation technologies and production of vigorous seedlings, three new populations as well as four ex situ germplasm collections of *N. yunnanensis* have now been established [7]. Phylogenetic studies of the six *Nyssa* species (*N. yunnanensis, N. javanica, N. sinensis, N. shangszeensis, N. shweliensis and N. wenshanensis*) recognized by the Flora of China have been conducted. Based on morphological and molecular evidence, the results suggest that only *N. sinensis, N. yunnanensis and N. javanica* should be recognized as species [8].

Recent advances in whole genome sequencing technology have provided valuable genomic resources to help us better understand the origin and evolutionary history of endangered species and to improve conservation strategies [9]. *Acer yangbiense*, another plant species with extremely small populations endemic to Yunnan Province, was sequenced in 2019. The *A. yangbiense* genome has a total length of 666 Mb with 13 chromosomes and a scaffold N50 size of 45 Mb [10]. The recently published genomes of *Nyssa sinensiss* and *Camptotheca acuminata* are the only two genome assemblies that have been sequenced within the Nyssaceae family. The *N. sinensiss* genome is 1,001.42 Mb in length with an N50 scaffold size of 3.62 Mb [11], and the *C. acuminate* genome is 403.17 Mb in length with an N50 scaffold size of 1753 Kb [12].

Although *N. yunnanensis* is not the first species sequenced in the Nyssaceae family, a detailed understanding of this endangered species’ genomic makeup along with other information, such as population structure and reproductive biology, is urgently required to improve the PSESP conservation strategy for its continued survival.

## Methods

**Figure 2.**
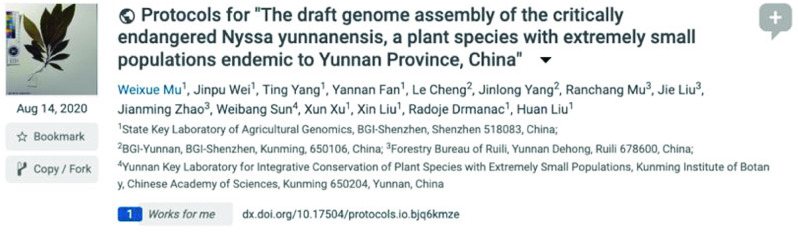
Protocol collection for the draft genome assembly of the critically endangered *Nyssa yunnanensis*, a plant species with extremely small populations endemic to Yunnan Province, China. https://www.protocols.io/widgets/doi?uri=dx.doi.org/10.17504/protocols.io.bjq6kmze

A protocol collection gathering together methods for DNA extraction and with DNBSEQ-G50 and 10X library construction and sequencing is available via protocols.io (Figure 2).

### Plant material

**Figure 3.**
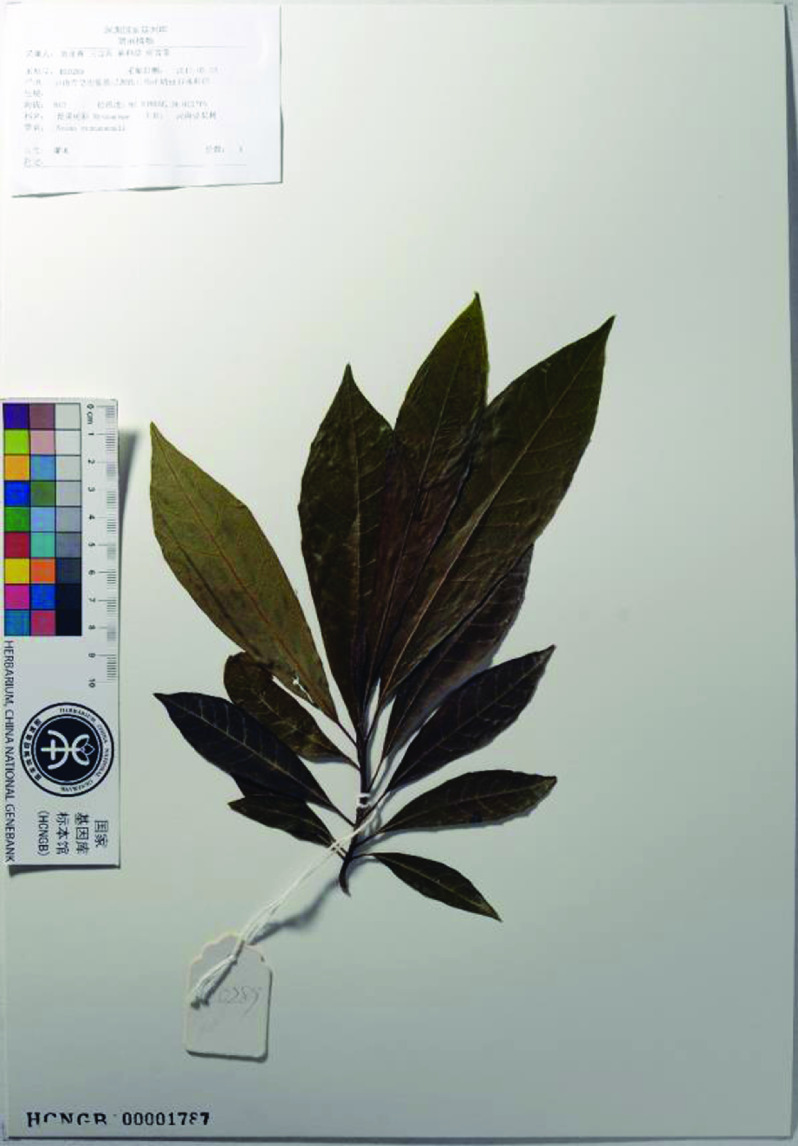
Photograph of the voucher specimens of *Nyssa yunnanensis*, stored in the CNGB herbarium (voucher RL0289).

We selected and sampled a 70 cm high individual tree of *Nyssa yunnanensis* from Ruili, Yunnan province, China (97° 56*′* 20.99*′′* N, 24° 03*′* 02.72*′′* E, altitude 843 m). Fresh young leaves were collected then immediately transferred into liquid nitrogen and stored in dry ice until DNA and RNA extraction. Voucher specimens and images were collected and stored in the CNGB herbarium (Figure 3). The extracted DNA is now stored in the BGI-sample center (voucher RL0289 and RL1182).

### DNA extraction and sequencing

Total genomic DNA was extracted from leaf tissues of *N. yunnanensis* using a modified CTAB method [13]. Quality control was done using a NanoDrop One UV-Vis spectrophotometer (Thermo Fisher Scientific, USA) and a Qubit 3.0 Fluorometer (Thermo Fisher Scientific, USA). A Sage Science Pippin Pulse electrophoresis system was used to evaluate the molecular weight of the DNA and high-molecular-weight (HMW) gDNA with a length of around 50 kb was obtained for further sequencing. The HMW gDNA was then loaded onto a Chromium Controller chip with 10X Chromium reagents and gel beads, and the rest of the library preparation procedures were carried out according to the manufacturer’s protocol [14]. Subsequently, the sequencing was performed on a DNBSEQ-G50 (previously known as BGISEQ-500, RRID:SCR_017979) platform at BGI-Shenzhen (BGI Co. Ltd., Shenzhen, China) according to the manufacturer’s instructions [15]. Using the whole-genome shotgun sequencing strategy, a total of ∼163.66 Gb of raw data (150 bp, paired-end) was eventually generated, covering about 100× the sequencing depth of the 1.64 Gb estimated genome size. All of the newly generated raw 10X Genomics reads were trimmed and filtered for adapter sequences and low-quality reads using Trimmomatic v. 0.38 (Trimmomatic, RRID:SCR_011848) [16] with the parameters “ILLUMINACLIP:adapter.fa:2:35:4 HEADCROP:5 LEADING:3 TRAILING:3 SLIDINGWINDOW:5:15 MINLEN:50”.

### RNA extraction and sequencing

Total RNA was extracted from young leaves of the same individual *N. yunnanensis* tree using a CTAB-pBIOZOL method [17]. The purity, concentration, and integrity of RNA samples were measured on a NanoDrop One UV-Vis spectrophotometer (Thermo Fisher Scientific, USA), a Qubit 3.0 Fluorometer (Thermo Fisher Scientific, USA) and with Agilent 2100 Bioanalyzer on-chip electrophoresis (Agilent Technologies, Inc; Santa Clara, CA) [18], respectively, to ensure that the samples qualified for transcriptome sequencing. The cDNA library was prepared using the TruSeq RNA Sample Preparation Kit v2 (Illumina, San Diego, CA, USA), and then sequenced on a DNBSEQ-G50 platform at BGI-Shenzhen (BGI Co. Ltd., Shenzhen, China), resulting in ∼8.85 Gb of raw transcriptome data (150 bp, paired-end). All of the raw sequence reads were further filtered using SOAPfilter v2.2 (SOAP, RRID:SCR_000689) with the parameters “-y -q 33 -i 200 -g 1 -M 2 -Q 20” to remove the adapters and low-quality reads.

### Genome size estimation

The 1.64 Gb genome size of *N. yunnanensis* was estimated using the 21 k-mer counts of clean reads from the 10X Genomics library. First, K-mer frequency distribution analyses were performed using the kmer_freq_hash software within the gce v1.0.0 package (GCE, RRID:SCR_017332) based on the clean 10X Genomics data with the parameters “-k 21 -l reads.lst -t 8”. Then, gce software within the same package was used to estimate the overall characteristics of the genome, including genome size, repeat proportions, and level of heterozygosity [19].

### *De novo* genome assembly

*De novo* assembly was carried out using Supernova v2.0.0 software (Supernova assembler, RRID:SCR_016756) [20] with the “–lanes =1 –localcores =48 –localmem =350 –maxreads 691040000” parameters. Raw 10X Genomics linked read data without trimming were used as the software recommended. Then, the gaps within the scaffolds were filled by GapCloser v1.12 (GapCloser, RRID:SCR_015026) [21] with the parameters “-l 150 -t 32” based on the clean 10X Genomics pair-end reads.

### Genome evaluation

The completeness of the *N. yunnanensis* assembly was estimated using two strategies. First, we assessed completeness using BUSCO (BUSCO; v3.0.2, RRID:SCR_015008) [22] with the parameters “-l Embryophyta_odb10 -m geno -c 8 -f”. Second, all the clean transcriptome reads and clean 10X Genomics reads were mapped back to the final genome assembly using BWA-MEM (BWA, version 0.7.16, RRID:SCR_010910) [23] with default parameters.

### Repeat annotation

Repetitive elements were identified using both homology-based and *de novo* predictions in the *N. yunnanensis* genome assembly. For homology-based prediction, RepeatMasker v3.3.0 (RepeatMasker, RRID:SCR_012954) and RepeatProteinMasker v3.3.0 [24] were applied with parameters “-nolow -no_is -norna -engine ncbi -parallel 1 -lib RepeatMaskerLib.embl.lib” and “-engine ncbi -noLowSimple -pvalue 0.0001”, respectively. The *N. yunnanensis* genome sequence was aligned against the known repeats database, Repbase v16.10 [25], at both DNA and protein levels to identify the known repetitive elements. For *de novo* prediction, RepeatModeler v1.0.5 (RepeatModeler, RRID:SCR_015027) [26] was first executed to build a *de novo* repeat library using the *N. yunnanensis* genome assembly with parameters “-engine ncbi -name mydb”. Then, RepeatMasker v3.3.0 [24] was employed to align the *N. yunnanensis* genome sequences against the *de novo* repeat library with parameters “-nolow -no_is -norna -engine ncbi -parallel 1 -lib final.library” to identify the repetitive elements. LTR_FINDER v1.05 (LTR_Finder, RRID:SCR_015247) [27] was used with parameters “-w 2 -s Athal-tRNAs.fa” for *ab initio* LTR retrotransposon finding and Tandem Repeats Finder v4.07 [28] was used with parameters “2 7 7 80 10 50 2000 -d -h” to identify tandem repeats.

### Gene prediction

The *N. yunnanensis* genome with repetitive regions masked was used to predict more genes. Protein-coding genes were predicted based on *de novo* prediction, homology search, and RNA evidence. For *de novo* prediction, Genemark-ES v4.21 (GeneMark, RRID:SCR_011930) [29] was used to carry out self-training with the default settings. To search for homologs, protein sequences of *Camptotheca acuminate* and *Arabidopsis thaliana* were used as references. For RNA evidence, a *de novo* approach was used. All of the clean RNA reads were assembled into inchworm contigs to function as expressed sequence tag evidence using Trinity v2.0.6 (Trinity, RRID:SCR_013048) [30] with the parameters “–min_contig_length 100 –min_kmer_cov 2 –inchworm_cpu 6 –group_pairs_distance 200 –no_run_chrysalis”. MAKER-P v2.31 (MAKER, RRID:SCR_005309) [31] was used to perform the prediction based on the evidence above. The first round of MAKER-P was run with the “protein2genome” and “est2genome” parameter set to “1” to obtain evidence-supported gene models. SNAP [32] was then applied to train these gene models. Then, MAKER-P was run for the second round with default parameters to generate the final consensus gene set. The search tool tRNAscan-SE v1.23 (tRNAscan-SE, RRID:SCR_010835) [33] was used for identifying tRNA genes. The rRNA sequences of *Arabidopsis thaliana* and *Oryza sativa* were BLAST against the *N. yunnanensis* assembly using BLASTN (BLASTN, RRID:SCR_001598) (E-value ≤ 1e−05) to identify rRNA genes. MicroRNAs and snRNAs were detected by searching the sequences against the Rfam database [34] using INFERNAL (Infernal, RRID:SCR_011809) [35] software.

### Functional annotation

The predicted gene models were further functionally annotated by querying the protein sequences against those in the public databases of Swiss-Prot [36], NCBI non-redundant (NR), KEGG [37], and TrEMBL with BLASTP (BLASTP, RRID:SCR_001010) with the parameters “-e 1e-05 -a 5 -m 8 -F F”. InterProScan v5.21 (InterProScan, RRID:SCR_005829) [38] was further used to search for the protein motifs and domains against public domain databases including the PFAM, PANTHER, PRINTS, PROSITE, ProDom, and SMART databases with the parameters “-goterms -f tsv -appl Pfam -appl PRINTS -appl ProSiteProfiles -appl ProSitePatterns -appl ProDom -appl SMART”.

## Results & Discussion

### Assembly and annotation of the *N. yunnanensis* genome

We assembled the draft genome assembly of the highly endangered tree species *N. yunnanensis* with DNBSEQ-G50 data from a 10X Genomics linked-reads library. The final genome assembly was 1.475 Gb in length, which is close to the estimated genome size of 1.64 Gb, with a scaffold N50 of 985.59 Kb and a contig N50 of 32.33 Kb (Table 1). The *N. yunnanensis* genome size we assembled was also close to the estimated genome size of 1.23 Gb based on the raw data produced [39] for the Digitization of the Ruili Botanical Garden project [40]. The GC content of the *N. yunnanensis* assembly was 42.18% excluding gaps, and a total of 54.24% of the assembly was composed of repetitive elements (Table 2). We ultimately obtained 39,803 protein-coding genes and successfully annotated 96.57% of the *N. yunnanensis* gene loci (Table 3). Non-coding genes were also annotated, identifying 175 microRNA (miRNA), 1,130 transfer RNA (tRNA), 1,502 ribosomal RNA (rRNA) and 3,106 small nuclear RNA (snRNA) genes (Table 4).

**Table 1:** Statistics of the *N. yunnanensis* genome assembly.

Parameters	Scaffold		Contig	
	Length (bp)	Number	Length (bp)	Number
Maximal length (bp)	17,928,324		636,521	
N90	3,639	38,762	2,907	67,003
N80	8,911	13,177	6,795	37,603
N70	27,779	2,699	12,643	22,971
N60	308,718	527	21,401	14,910
N50	985,593	280	32,329	9,853
N40	1,699,409	166	4,5737	6,370
N30	2,587,311	96	62,625	3,875
N20	3,773,602	49	86,821	2,057
N10	6,183,248	17	127,405	771
Total length (bp)	1,474,960,449		1,330,978,457	
Number ≥ 100bp		288,519		320,818
Number ≥ 2000bp		57,709		79,919
Percentage of N content		9.76%		

**Table 2:** Statistics of repetitive sequences identified in the *N. yunnanensis* genome.

Category	Total repeat length (bp)	% of assembly
DNA	133,291,367	9.04%
LINE	47,813,283	3.24%
SINE	1,053,993	0.07%
LTR	690,959,337	46.87%
Tandem repeats	6,108	0.0004%
Unknown	135	0.000009%
Combined	799,507,629	54.24%

Note: DNA: DNA transposon; LINE: long interspersed nuclear element; SINE: short interspersed nuclear elements; LTR: long terminal repeat.

**Table 3:** Summary of protein-coding genes annotated in the *N. yunnanensis* genome.

Characteristics of protein-coding genes	
Total number of protein-coding genes	39,803
Mean gene size (bp)	2576.05
Mean CDS length (bp)	957.64
Mean exon number per gene	4.16
Mean exon length (bp)	230.26
Mean intron length (bp)	512.32
Functional annotation by searching public databases	
% of proteins with hits in Swiss-Prot database	76.01
% of proteins with hits in NCBI nr database	96.30
% of proteins with hits in KEGG database	72.15
% of proteins with hits in TrEMBL database	95.90
% of proteins with hits in Interpro database	70.99
% of proteins with functional annotation (combined)	96.57

**Table 4:** Statistics of predicted ncRNA in the *N. yunnanensis* genome.

Type		Number	Average length (bp)	Total length (bp)	% of genome
**miRNA**		175	120.8629	21,151	0.0014
**tRNA**		1,130	75.2221	85,001	0.0058
**rRNA**	**rRNA**	1,502	142.5366	214,090	0.0145
	**18S**	469	231.5117	108,579	0.0074
	**28S**	458	106.0764	48,583	0.0033
	**5.8S**	116	111.0603	12,883	0.0009
	**5S**	459	95.9586	44,045	0.0030
**snRNA**	**snRNA**	3,106	108.3973	336,682	0.0228
	**CD-box**	2,911	106.8636	311,080	0.0211
	**HACA-box**	51	129.3333	6,596	0.0004
	**splicing**	144	131.9861	19,006	0.0013

### Data validation and quality control

The BUSCO analysis showed that up to 1244 (90.5%) of the expected 1375 conserved plant orthologs were detected as complete in the *N. yunnanensis* assembly and 81.9% of them were identified as complete and single-copy genes (Table 5). The RNA mapping showed that 98.95% of the reads could be successfully mapped back to the assembled genome and 83.74% of them were properly paired. The DNA mapping resulted in a 96.88% mapping rate and 86.74% of them were properly paired. These results demonstrate the high completeness of the *N. yunnanensis* assembled genome.

**Table 5:** BUSCO assessment of *N. yunnanensis* genome.

BUSCO benchmark	Number of genes	Percentage
Complete BUSCOs	1244	90.5
Complete and single-copy BUSCOs	1126	81.9
Complete and duplicated BUSCOs	118	8.6
Fragmented BUSCOs	63	4.6
Missing BUSCOs	68	4.9
**Total**	1375	/

### Potential for reuse

Here we report a draft genome assembly of the PSESP plant species *N. yunnanensis*. The completeness assessment carried out by reads mapping and BUSCO assessment indicated the high completeness of this draft assembly. As part of the 10KP (10,000 Plants) Genome Sequencing Project [41], the sequencing data and the well-annotated draft assembly generated in this study can be used for future phylogenetics and comparative genomics analyses, such as resolving the controversial phylogenetic relationships within the Nyssa genus. In particular, due to the extremely small population structure of *N. yunnanensis*, the genomic resources released in this study will support further research on the conservation biology of this highly endangered species as well as other PSESP species.

## Data Availability

The 10X Genomics clean reads and RNA-seq clean reads are deposited in NCBI under the BioProject accession PRJNA438407, with SRA accession number SRX8345787 and SRX8373586. These reads are also deposited in the CNGB Nucleotide Sequence Archive (CNSA) with accession number CNP0001048. Genome assembly, protein-coding genes, and repeat annotations are deposited in the *GigaScience* GigaDB repository [42].

## Abbreviations

10KP: 10,000 plant genome project; BUSCO: Benchmarking Universal Single-Copy Orthologs; CR: Critically Endangered; CTAB: cetyl-triethylammonium bromide; HMW: high-molecular-weight; PSESP: Plant species with extremely small populations;

## Subject areas

Genetics and Genomics; Plant Genetics, Botany
